# Why Migrant Workers in Northeast Thailand Lack Adequate Dental Services: A Confluence of Instrumentality

**DOI:** 10.3390/healthcare14193157

**Published:** 2026-09-23

**Authors:** Soraya Chaleoijit, David Hughes

**Affiliations:** 1 Sirindhorn College of Public Health Khon Kaen, Faculty of Public Health and Allied Health Sciences, Praboromarajchanok Institute, 90/1 Anamai Rd., Naimuang Sub-District, Muang District, Khon Kaen 40000, Thailand; soraya@scphkk.ac.th; 2 Faculty of Medicine, Health & Life Science, Swansea University, Glyndwr Building, Singleton Park, Swansea SA2 8PP, UK

**Keywords:** Thailand, migrant workers, migrant employment, oral health, dental services, public health insurance

## Abstract

**Background:** Despite Thailand’s reputation for universal health coverage and ASEAN’s stated commitment to expand regional social protection, migrant workers in the kingdom experience poorer access to dental care than Thai citizens. This paper presents qualitative findings on dental provision collected in 2016–2017, which, given the largely unchanged institutional and policy framework affecting migrant dental services, may help explain why care remains limited, uneven, and under-prioritised. While we cannot be sure that behaviour and practices affecting dental provision remain the same nine years later, recent studies of migrant worker access to general healthcare suggest that they are unlikely to have changed greatly. Methods: The research involved 61 interviews with policymakers, administrators, dental professionals, and employers, alongside 11 focus groups completed with migrant workers. Results: Access to dental care was constrained by structural barriers: complex registration pathways, disconnected multiple insurance schemes, and employer non-compliance. However, these factors alone did not fully explain sub-optimal service utilisation. Although there was variation in opinion within each stakeholder group, perspectives were typically characterised by instrumental reasoning: calculations of cost, convenience, and perceived need. Many policymakers viewed existing arrangements as a good-enough interim solution pending eventual enrolment in the Social Security Scheme; most dental professionals framed migrants as low-value patients with limited entitlements; employers weighed administrative burden and worker turnover when deciding whether to fund insurance enrolment; and migrant workers themselves frequently delayed care, normalised dental pain, or sought cheaper treatment across the border. These intersecting orientations formed a “confluence of instrumentality” and collectively suppressed demand for reform, keeping dental care peripheral within migrant health policy. Conclusions: Although the delay in publishing our data is a major limitation, the findings of the study provide potentially valuable insights into the present situation. Everyday reasoning among key stakeholders can reproduce and accentuate structural inequities. Policymakers need to address both systemic constraints that affect migrant access to dental care and the pragmatic calculations that mediate how such constraints affect utilisation.

## 1. Introduction

Despite Thailand’s reputation as a pathfinder in implementing universal health coverage [1] and ASEAN’s stated aspiration to strengthen its socio-cultural community “pillar” [2], migrant workers continue to face sub-optimal and inconsistent access to dental services. Thailand is a major destination for unskilled workers from neighbouring ASEAN countries, mainly Cambodia, Lao PDR, Myanmar and Vietnam. Drawing on qualitative data on dental services for migrant workers collected in 2016–2017, this paper discusses various factors that help explain why dental care remains inadequate. The study found that dental provision for migrant workers was worse than that available to Thai citizens, as it still is today. This gap highlights both the limitations of Thailand’s much-heralded universal coverage healthcare system [3] and the limited progress made toward ASEAN’s goal of providing basic social protection across the region. While recent reviews show that a substantial evidence base exists on migrant health services more generally [4,5], there is a paucity of research on access to dental services.

This paper argues that sub-optimal dental provision cannot be explained solely by the reluctance of government and key institutions to respond to demands from NGOs and civil society organisations (CSOs); an important factor is that professionals and supposed beneficiaries often express ambivalence about the need for improved care. Based on the accounts of policymakers, administrators, professionals, employers, and migrant workers, we identify an instrumental orientation—a pattern of reasoning in which decisions are shaped by calculations of cost, convenience, and perceived need. Although these orientations differ across stakeholder groups, they combine to downplay the urgency of reform and reduce pressure for political action. We conceptualise this dynamic as a *confluence of instrumentality*, in which the intermeshing attitudes of key actors limit the priority given to dental services within the wider policy landscape.

Thailand’s rapid economic growth in the 2000s, following the 1997 Asian financial crisis, intensified demand for low-waged migrant labour. Immigration from neighbouring countries increased sharply, driven by poverty in origin countries and Thai labour shortages in sectors where Thai citizens were unwilling to work. The Migrant Working Group [6] reported that by April 2023 more than 2.49 million migrants from Laos, Cambodia, Myanmar, and Vietnam held work permits in Thailand, a figure that did not include the substantial number of undocumented workers. Most migrants are employed in unskilled “3D jobs”—dirty, difficult, and dangerous—in construction, agriculture, fisheries, livestock husbandry, stevedoring, mining and quarrying, manufacturing, domestic work, and the entertainment sector (including sex work).

One major limitation of this report concerns the delay that has occurred in moving from a doctoral thesis submitted in 2020 [7] to a focused research paper, something which may raise doubts about the relevance of our commentary on the contemporary situation and policy framework. However, perhaps surprisingly, the institutional and regulatory framework affecting migrant healthcare remains largely unchanged. The rules concerning legal entry for pre-arranged employment, the procedure for regularising the status of migrants who arrive without prior approval, and the continued presence of significant numbers of migrants working illegally, all remain substantially the same as at the time of our fieldwork. The dental packages offered under different public insurance schemes stayed unchanged until May 2026, when both eligible Thais and migrant workers benefitted from a relaxation of the annual treatment cost ceiling applied in the Social Security Scheme (SSS). This slow pace of change reflects the politically controversial nature of policy on migrant labour and difficulties in addressing the shortcomings of the present arrangements.

In recent years, disquiet about deficiencies in healthcare for migrant workers has only intensified. Against the backdrop of the ASEAN Economic Community’s efforts to strengthen social protection within the region, and Thailand’s status as a leader in implementing universal healthcare coverage, NGOs and CSOs have pressed the Thai government to improve coverage for migrants [8]. However, dental services remain largely absent from these debates, despite evidence that dental pathology can cause significant discomfort, contribute to cognitive deficits and fall risks in older adults [9], and facilitate early detection of other disease conditions [10].

Thailand’s only formal legal pathway for unskilled migrant workers from Laos and Cambodia remains the bilateral Memoranda of Understanding (MoU) on labour cooperation that were agreed shortly before our fieldwork began. In the case of Lao PDR, a MOU was signed in 2015 and updated in 2017, while the MOU with Cambodia was agreed in 2016. The latter was renegotiated in 2025 to allow simplified recruitment and introduce greater flexibility for seasonal/short-term work but otherwise remains substantially unchanged. The original MOUs were valid for four years but were then extended without significant changes. After verification of employment, employer status, identity, and general health, MOU migrants receive a non-Thai ID card valid for 4 years and entitlement to enrol in the SSS, the public insurance scheme for Thais working in the formal employment sector. The SSS provides comprehensive healthcare coverage, including dental services [11,12]. However, studies show that only around half of eligible migrants enrol, in part due to a waiting period before coverage starts and reluctance to accept wage deductions [1,13].

None of the migrants working for employers participating in the study or included in the focus groups had entered via the MoU route. Instead, they arrived as undocumented migrants and later sought to regularise their status through a programme introduced by the government to manage the problem of non-MOU arrivals. Such workers are directed to One Stop Service Centres (OSSCs), open during limited government registration windows. There they are required to pay a fee, produce proof of employment and documents needed for nationality verification (NV), and undergo a medical examination to screen for communicable disease. Successful applicants receive a non-Thai ID card, a temporary work permit (“pink card”), eligibility to enrol in the Migrant Health Card Scheme (MHCS) at extra cost, and permission to remain while awaiting NV. At the time of the study, only a minority of OSSC registrants took up the option to enrol in the MHCS [11,14], although this rose to around half by 2025 [1]. The MHCS has been described as the Health Insurance Card Scheme or the Health Insurance Scheme for Foreigners in some other publications.

NV can only be completed in a few designated centres, all distant from the northeast and requiring long travel and additional costs. Once NV is completed, workers may enrol in the SSS, but many prefer to remain uninsured or continue with the MHCS, which can be renewed annually (or biennially in some provinces). Large numbers of migrant workers therefore remain undocumented or rely on the pink card for extended periods without full documentation. Those seeking healthcare, including dental care, mostly pay out-of-pocket at public or private facilities. The study focus group participants included both documented and undocumented workers.

Although both the SSS and MHCS offer basic dental services with no or low user fees (30 THB, approximately £0.70 GBP), the benefit packages differ. Both schemes cover fillings, extractions, and scaling. However, x-rays and oral medications potentially covered under the SSS have in practice been provided only at the discretion of providers, and only the SSS covers dentures. In comparison, Thai citizens, outside the formal employment sector and covered by the Universal Coverage Scheme (UCS), are eligible for preventive care and some specialist procedures not available under the MHCS.

The limited changes in this framework that have occurred between 2017 and 2026 are listed in Table 1. These do not greatly change the pathways and entitlements described above.

## 2. Materials and Methods

The research questions identified for the study concerned the perspectives of multiple stakeholders at different levels of the healthcare system on dental healthcare needs, the perceived pressure on services, the problems encountered, and the experience of accessing dental care. These questions touched upon the subjective perspectives of key participants, which suggested the need for a qualitative approach. Consequently, it was decided to employ a combination of in-depth interviews and focus groups. Interviews were conducted with policymakers, administrators, dental professionals, and employers, while focus groups were completed with Laotian and Cambodian migrants who were potential service users. Focus groups represented an economical way of engaging with around 40 migrant workers in a single fieldworker study that would also involve over 60 interviews with other stakeholders. It was anticipated that migrant workers would feel more comfortable in a small group of peers than in individual interviews, and that interactions in group discussions were likely to generate richer insights than would be forthcoming from vulnerable people who might be inhibited in a one-to-one situation.

Fieldwork was undertaken in five Thai provinces bordering Lao PDR and Cambodia. The provinces adjacent to Lao PDR comprised Ubon Ratchathani and the three designated special economic border zones of Nong Khai, Nakhon Phanom, and Mukdahan, while Surin province borders Cambodia. These provinces are predominantly rural, with each containing a medium-sized provincial city.

The study was conducted in accordance with the principles of the Declaration of Helsinki. Participants were supplied with a study information sheet and gave written informed consent to participate and for anonymised data to be reported in research papers and reports. Ethical approval for the study was obtained in both the United Kingdom and Thailand. The Swansea University Research Ethics Committee granted approval on 19 May 2016 (Ref. no. 200516), followed by approval from the Research Ethics Committee of the Ministry of Public Health (MoPH) Provincial Health Office in Nong Khai on 28 October 2016 (Ref. no. 17/2016).

The composition of the four main stakeholder groups is shown in Table 2:

(a) Policymakers and administrators: Eleven key informants were recruited from the MoPH, the National Health Security Office (NHSO), three NHSO regional offices, and five MoPH Provincial Health Offices (PHOs). Two policymakers with expertise in immigration policy were based within the MoPH’s Bureau of Policy and Strategy and affiliated with the International Health Policy Programme (IHPP). Two informants held posts in the NHSO headquarters in Bangkok, the agency responsible for managing Thailand’s Universal Coverage Scheme (UCS), and two others in NHSO regional offices in the northeast. Five informants were based in PHOs, where they were responsible for overseeing the administration and performance of MoPH facilities within the five study provinces. Specific job titles are omitted to preserve participant anonymity, not least because some respondents held very senior positions.
(b) Professionals: Interviews were conducted with 23 dental professionals employed by the MoPH in district hospitals and clinics, alongside six private dental practitioners spread across the study provinces.
(c) Employers: A total of 21 employers across the five provinces were interviewed. These participants operated in sectors employing migrant labour, including construction, manufacturing, retail, hospitality, transport, and agriculture. Employers were typically responsible for facilitating worker registration and arranging access to healthcare services.
(d) Migrant workers: Two focus groups were planned for each province, but in the event a third focus group was conducted in one province because of the poor quality of the data collected. Migrant worker participants were recruited via employers (total n = 37). Fourteen of these had not yet registered and were therefore working illegally. Seven crossed the border daily to work for a Thai employer in a market fruit business on the Thai side.

As is common in qualitative studies, sampling was based largely on convenience, with purposive selection where possible. Even if a survey design had been employed, it would have been infeasible to assemble traditional probability samples of employers and workers, because of the number and diversity of work sites and the well-documented problems of reaching a vulnerable migrant population [15]. Policy makers and senior administrators were recruited based on their specialisms and aided by contacts in the MoPH and NHSO known to DH through previous research and consultancy. PHO respondents were identified based on location and their professional roles, and were approached sequentially until the desired sample size was achieved. The sample of public-sector dental personnel (15 dentists and eight dental nurses) was assembled to ensure approximately equal representation across provinces, with at least four respondents per province. Six private dentists were included, distributed across the provinces, with two located in the largest border city.

Employers were recruited through convenience sampling, with efforts made to ensure a spread of geographical locations and business types (see Table 3).

Migrant workers are a notoriously hard-to-reach group. In rural Thailand, there were no CSOs that could direct us towards potential participants, and such workers make little or no use of social media networks that have been used to aid recruitment in some Western countries. Because of discrimination, stigmatisation and irregular status, such workers tend to keep a low profile and may be wary of contact with what might be perceived as research connected to official bodies. We settled on the less-than-perfect recruitment strategy of taking participating employers as the initial point of contact, seeking employer agreement and then allowing workers to decide whether to take part. In a study where employer/employee relationships were an important topic, this had the advantage of allowing us to triangulate data from both sides, in some cases providing contrasting perspectives on the same practices or events. However, since employers could refuse to allow their workers to participate, this brought the risk of “gatekeeper bias” and under-representation of work sites with poor employment practices. We had no budget to offer monetary incentives for participation, and the only reward provided was a free dental care kit. Strategies involving forms of “snowball” or “chain” sampling, such as the “respondent-driven sampling” utilised in some migrant worker survey research [15], involve assembling a sample for a series of interviews over time as one respondent suggests others. This would be infeasible in a focus group study. Where the employers interviewed were unwilling for their workers to participate, similar businesses were approached to gain permission to recruit the required number of focus group participants (see Table 4), albeit with no corresponding employer interviews.

The need for participant consent may have introduced additional selection bias. Concerns about legal status and discrimination, together with the time required to participate, may have discouraged some migrant workers from taking part and thereby reduced participation rates. Consequently, we were able to recruit only three or four participants per focus group, rather than the planned four to six. In Nakhon Phanom province, one focus group comprising three young male participants with minimal education yielded only limited data due to guarded responses. To collect richer data, an additional focus group was conducted in this province, resulting in a total of 11 focus groups. Where hard-to-reach populations are involved, pragmatic judgements must be made about what constitutes good-enough data saturation. We felt that adequacy was best assessed by the relevance and interest of the data collected and corroboration of findings by other stakeholders, which we judged to be acceptable. Social desirability and disclosure biases will be issues in any study of a vulnerable population. This does not necessarily render such data invalid but limits the inferences that can be drawn. What participants say can legitimately be used to examine perspectives and identify themes and mechanisms, but will not reliably indicate prevalence of reported practices or accurate disclosure of illegal behaviour.

All prospective participants were provided with an information sheet with details of the study, including contact details for the lead author. Informed consent forms were completed for all participants, confirmed with a signature or thumbprint. For participants with limited literacy, study information was communicated verbally.

The qualitative interviews were based on outline interview guides tailored to each participant group, incorporating open-ended questions. These guides allowed for flexible lines of questioning, enabling the inclusion of additional probes where necessary. Focus group discussions followed a similar structure. The guides were informed by the literature review and refined following two pilot studies involving volunteer participants who were not included in the final sample. Focus groups with Laotian participants were conducted in the Thai-Isan dialect, in which the lead author is fluent, while those involving Cambodian participants were conducted mainly in Khmer with the assistance of a bilingual dental nurse experienced in treating migrant workers, who acted as translator. The focus groups were conducted in quiet settings near the place of employment, such as small eateries, a private room in a restaurant, and a largely unused seating area in a market, all with no employer present. SC and the translator managed the two focus groups involving Cambodian workers, while SC alone conducted the other nine focus groups.

All interviews and focus groups were audio-recorded with prior permission, and fully transcribed in Thai. Transcribed data were organised and analysed using a generic content analysis approach [16,17], adapted to the context of the study, and described in detail in SC’s doctoral thesis [7]. The analytical process involved categorising textual data into themes and subthemes and attaching codes that enabled the identification of overarching patterns and variations in responses. As a first stage, transcripts were read and clusters of themes and subthemes with associated codes were identified for each of the main stakeholder groups. This was undertaken using the qualitative data analysis application MAXQDA, version 2018. Preliminary coding was undertaken by SC, with DH checking and suggesting possible adjustments. As analysis proceeded, SC built up a taxonomy of codes and created a codebook, together with a provisional mapping of how the codes fitted together. Advice was taken from DH to ensure that the categories fitted together logically and were sufficiently comprehensive. Theme generation had both deductive and inductive elements. Certain themes such as the working of the OSSC regularisation process and uptake of public insurance were suggested by existing literature, while others such as stakeholder accounts of specific operational problems and how they were managed emerged from participants’ accounts. Passages selected as possible data extracts were translated into English. The validity and reliability of the analysis are discussed in the thesis [7]. Regarding the craft of fieldwork, we paid particular attention to multi-participant corroboration of key findings, prioritising the accounts of those in a position to know about a given issue, and triangulating accounts offered from different stakeholder standpoints.

This is essentially the analysis that informs both the thesis and the present paper. The paper narrows the focus and attempts some additional theoretical development. The thesis had been concerned partly with the impact of the strengthening of the ASEAN Economic Community and whether the development of a community socio-cultural pillar was improving social protection for migrant workers entering Thailand. The differing and sometimes overlapping perspectives of different stakeholders were mapped out and their intersecting influences discussed. However, in the thesis the concept of instrumentality was applied primarily to policy makers, taking account of the military government’s tendency to alternate between welcoming migrants when labour was needed and forcing them out when numbers were seen as excessive, as happened in 2014 and 2017. The perspectives of employers and dentists were described as transactional, while migrant workers were seen to cope with sub-optimal public dentistry by, for example, finding alternative paths to treatment or delaying treatment. In looking again at the main themes in our qualitative data, we concluded that instrumentality was in fact apparent in the behaviour of all the main stakeholder groups and that, although instrumental reasoning took different forms, the underlying attitudes meshed together to reduce the perceived need for change. This was how we arrived at the notion of the confluence of instrumentality presented here.

Given the political sensitivity of migrant healthcare and the hard-to-reach nature of migrant workers, the perceived identity of the researchers—a student and supervisor—will almost certainly have affected respondent participation and how participants constructed their answers. When planning the research SC was already an experienced dental practitioner, who owned a multi-site dental practice in Khon Kaen and lectured on dentistry at the major MoPH training college in Isan region. She had good contacts with professional colleagues in Isan region that assisted with the recruitment of respondents. SC was aware from her own dental practice that delivery of services to migrant workers was not working well and was sympathetic to their situation. However, she had not been involved in public debate on migrant healthcare and had no fixed ideas about how policy should best be developed. SC was well aware that her interactions with migrant workers would probably be affected by a perceived gap in social class, and tried to dress and behave in ways that minimised any impression of what Thais term “hi-so” status.

While a visiting professor at Mahasarakham University in the early 2000s, DH had led a major study of the implementation of the universal coverage reforms in three northeastern provinces [3,18]. Later he was recruited by the MoPH to the Thai team that provided background support for the 10-year assessment of the UCS [19] and was involved in other ongoing Thai research. In 2014. he published an overview of Thailand’s healthcare system co-written with the then NHSO Deputy Secretary General, which was updated in 2025 [20]. Hughes’s contacts in the MoPH and NSHO helped facilitate access to senior informants and support for the study, but he had no face-to-face contact with study participants during the fieldwork period. None of DH’s previous studies had been concerned directly with migrant healthcare and prior to this study he had no firm views about the best direction for policy development.

## 3. Results

### 3.1. Policymakers and Administrators

Policymakers and senior administrators interviewed can be broadly categorised into two groups: those sympathetic to the situation of migrant workers and those who viewed migrants primarily as a public health risk and a burden on health services. For most informants, the main concern centred on the overall healthcare package available to migrants, with dental services perceived as a comparatively low-priority component of a wider structural issue.

Negative perceptions of migrants were frequently articulated, including claims about the risks of communicable disease transmission, human trafficking, prostitution, crime, and fiscal pressure on public services. Particular concern was expressed about vaccine-preventable diseases—such as diphtheria, pertussis, tetanus, and poliomyelitis—with one respondent attributing a recent disease outbreak in a border province to incoming workers. The fact that most migrants worked in so-called “3D jobs” was taken as an indication of low social status and contributed to such attitudes.

Although many informants acknowledged that dental services available to migrants were less comprehensive than those provided for Thai citizens, only a minority considered this inequity problematic. Limited access to care was generally framed as an issue affecting those who had yet to regularise their legal status and enrol in the SSS. This group included undocumented workers employed by non-compliant employers and persons with pink cards still midway through the regularisation process. Some policymakers and employers suggested that many migrants eligible for SSS membership opt to remain uninsured, often because of employer reluctance to pay SSS contributions:

“If we recognise migrant workers as human beings, they should be enrolled in the health insurance system… Why do employers not contribute to the SSS?… They have never considered that workers need care.”

(A2, IHPP)

A recurring theme in the interviews was that the MHCS was a temporary solution, which in time would be absorbed into the SSS. While several respondents advocated direct enrolment and making what was a voluntary scheme mandatory, others questioned whether it was worth re-jigging what was an interim arrangement. Low uptake—particularly among undocumented and highly mobile workers—was identified as a serious limitation, resulting in a disappointingly small risk pool:

“As a voluntary scheme, it fails to attract sufficient participation to ensure effective risk-sharing.”

(A1, IHPP)

While some respondents argued that itinerant workers required only a basic package of care, others talked about the need for equity and Thailand’s aspiration to develop an UHC scheme that would stand as a global exemplar. These informants framed migrant healthcare as a matter of human rights and highlighted reputational risks associated with labour exploitation, including Thailand’s placement on the US Department of State’s Trafficking in Persons Report Tier 3 watch list in 2014:

“We should not treat them as second-class citizens… We need to improve welfare, at least on the health side.”

(A5, PHO)

“We need to ensure a healthy labour force… Our international reputation is at stake.”

(A1, NHSO)

Those taking this view argued that the MHCS should be expanded to align more closely with entitlements available to Thai citizens. The issue of equal treatment is particularly salient in dentistry, where disparities in provision remain. Notably, the MHCS excludes preventive dental care, even for children, despite its long-term benefits. These limitations were attributed to financial and human resource constraints within the MoPH:

“Dental benefits are determined by available resources… Only basic treatments are covered.”

(A2, IHPP)

Views diverged regarding the adequacy of these restricted entitlements. While a few informants mentioned possible improvements to the MHCS, a prominent theme was that the present arrangements were acceptable given that workers would eventually transition to the SSS:

“Basic dental services are sufficient… More complex treatments can be obtained privately.”

(A11, PHO)

Some administrators further argued that migrants should not receive better benefits than Thai citizens got. One informant proposed introducing a cost ceiling for MHCS dental treatments, analogous to the 900 baht annual limit under the SSS:

“The MHCS dental benefit should have a similar ceiling… even 500 baht might be sufficient.”

(A7, PHO)

In contrast, respondents more sympathetic to migration emphasised the importance of preventive care and parity:

“It is unreasonable to exclude migrants from basic dental benefits available to Thais.”

(A6, NHSO)

Concerns were also raised regarding the attitudes of service providers to migrants. While policy initiatives promoting “migrant-friendly” services—such as translation support and multilingual materials—were acknowledged, informants said that linguistic and cultural barriers persisted. To address low enrolment, some informants proposed that MHCS membership be made mandatory for all migrants completing OSSC registration:

“If entry were conditional on purchasing health insurance, full coverage could be achieved… though affordability remains a constraint.”

(A11, PHO)

These findings should be interpreted within the broader political context. During the study period the military government tightened the regulations affecting migrants. Previous accounts of the Thai UCS reforms suggest they were driven by socially liberal bureaucratic reformists within the MoPH and National Health Security Office (NHSO), whose perspectives were not always shared by politicians or staff working in the healthcare system [21,22]. Tensions persisted during the research period with some conservative politicians advocating increased user charges and curtailed benefits, even for the mainstream Thai UCS.

Overall, although policymakers and administrators differed in the importance they attached to migrant rights, most tended to view migrant health policy through an instrumental lens. Some portrayed the migrant workforce as a transient population that could be increased in times of labour shortage and reduced when numbers were judged excessive, meaning that long-term comprehensive healthcare provision was unnecessary. Even those who favoured equitable provision mostly viewed the MHCS as an interim arrangement that could be wound down as workers enrolled in the SSS, so that comprehensive system change was unnecessary.

### 3.2. Professionals

Most dental professionals interviewed characterised migrant workers as a patient group associated with low reimbursement rates, limited treatment coverage under the MHCS, and a reluctance to utilise either the SSS or MHCS even when eligible. Respondents frequently described migrants as hesitant to pay for private dental services. Overall, there was little support among dental personnel for expanding existing public insurance programmes or entitlements. Some interviewees nevertheless proposed incremental improvements, such as incorporating a dental check-up into the MHCS screening process and reducing differences in entitlements between the SSS and MHCS.

Consistent with the views expressed by several senior administrators, some dental professionals articulated negative views of migrants that reflected stereotypes circulating in the wider population. Interviewees commonly commented on migrants’ personal hygiene, lack of health knowledge, and supposed unwillingness or inability to pay for services. Migrant workers were widely viewed as a disadvantaged population vulnerable to exploitation, human trafficking, precarious employment, and destitution. As one dentist stated, “The view of them is generally negative. Since they are a cheap labour, their quality of life has not been accepted. Their illnesses and crime are a burden for the government” (D3).

Several respondents expressed the belief that migrant workers should not receive the same benefits as Thai citizens. One dentist questioned the rationale for extending MHCS benefits to Laotian workers, stating:

“I don’t understand why they (the government) allow Laotian workers to have the same benefits under the MHCS as Thais get with the [UCS) gold card. My personal thinking is… I think they are probably yearly labour so they can’t join the SSS”.

(D8)

Another respondent framed the issue in terms of resource allocation:

“I think it [public insurance] should support Thais more than these. Like we lose the benefit because of the burden of those migrant workers… But we waste it. Anyway, the other view is that we have to see this as a human right”.

(D11)

Several informants argued that the primary barrier to care was not the shortcomings of government support programmes but migrants’ unwillingness to use them. One dentist described a case in which a young migrant worker repeatedly delayed treatment despite being fully entitled under the SSS:

“Once the biopsy process was started and the operation date was set, he said he had to visit his family in Lao PDR… Finally he returned to me with the infection [having become more serious] as I had said might happen”.

(D10)

Dental staff identified several barriers to access, including treatment costs, the reluctance of younger migrants to pay for insurance, and the complexity of enrolment rules for both the SSS and MHCS. Respondents noted that newly arrived migrant workers often lacked the financial means to pay the MHCS fee or to enrol independently in the SSS. Employers were generally expected to pay the initial costs and subsequently deduct these from wages. One dentist suggested that the employer contribution made the SSS more attractive to migrants: “I think the migrant workers want to join the SSS rather than MHCS because their employer contributes money to the scheme” (D8). However, many employers were reportedly unwilling to cover these costs, and workers themselves often resisted wage deductions.

Several respondents attributed low enrolment to migrants’ perceptions of value for money. As one dentist explained:

“If I bought the health insurance and only use it rarely in the year, I might feel that I don’t want to buy it next year… It is not worth it. Why should I buy a new one? For migrant workers, their real problem is occupational accidents”.

(D1)

Others suggested that younger migrants in particular were reluctant to pay for insurance, preferring to spend their income on consumer goods such as mobile phones (D1; D8).

Two hospital-based dentists reported that many migrants were unable to pay for treatment. In such cases, patients were referred to administrative staff to assess entitlements or negotiate payment arrangements. One dentist noted that the hospital sought to discourage any belief that treatment was free without MHCS or SSS entitlement: “We aim to let them know that it is not easy to get free service. Otherwise, other Laotian patients will congregate in the hospital” (D1).

For more costly procedures not covered by insurance, such as root canal treatment, instalment plans were sometimes offered, though uptake was reportedly low.

“Some of them came here with severe dental pain caused by a big cavity, but they just wanted a filling. However, I suggested that the real treatment needed was root canal treatment. I suggested this kind of treatment, but I also informed them that an extra bill must be paid. I talked to the patient. It seems they aren’t ready to pay for this. Some of them considered the treatment but they seemed to struggle to get the money. Actually, they can pay by instalments for root canal treatment. I explained to them about it but they still couldn’t afford the bills”.

(D11)

Respondents also highlighted practical barriers to care, including migrants’ struggles to attend scheduled appointments and understand hospital procedures. Many migrants were unable to attend appointments due to employer restrictions and were unfamiliar with the requirement to register and verify entitlements before receiving treatment.

“One problem is that they don’t know the hospital process. They sometimes head to the dental department without registering to establish their rights. They need to check their entitlements before getting a service. Regarding the appointment time, it is a problem. Sometimes they can’t come to the hospital at a specific appointment time if their employer doesn’t allow them to.”

(D1)

It became apparent that dental staff themselves often lacked full knowledge of the rules governing the MHCS and SSS. Several believed that the MHCS provided the same benefits as the UCS for Thai citizens and were unaware of the differences in entitlements. One dentist was uncertain whether a benefits maximum applied to MHCS beneficiaries.

While professionals’ perspectives on the migrant workforce varied, most viewed migrant workers as low-value patients whose processing caused complications and who often failed to comply with treatment recommendations. The concerns expressed in the interviews tended to centre on rectifying problems like procedural complexity and low reimbursement ceilings that affected dentists rather than equitable treatment for migrants.

### 3.3. Employers

In the employer interviews, the issue of migrant workers’ dental care was overshadowed by broader concerns about the cost and complexity of the OSSC registration and nationality verification processes, worker retention, and the practicalities of arranging general healthcare. Like health professionals, employers generally perceived migrant workers as economically disadvantaged individuals with limited access to healthcare services in their home countries. These workers were widely understood to come to Thailand for instrumental reasons, most commonly to support extended families engaged in subsistence agriculture in rural villages—a pattern familiar to many northeastern Thais.

Employers tended to categorise migrant workers into two groups: those likely to remain in employment for an extended period and those who work only briefly in the northeast before moving on. This distinction shaped employers’ willingness to invest time and resources in assisting workers with healthcare arrangements. Many workers were perceived as falling into the latter category:

“Some workers just work for a few days and give up. These were mostly new arrivals. We paid for them, but they were soon gone. It was like that. So we will consider the worker. Do they really want to work?”

(E13)

“My workers usually work with me for no more than six months and quit the job without notice. Most are teenagers so they are not patient people. They will change their job if they are bored.”

(E9)

Nevertheless, employers also described a subset of migrant workers from both Laos and Cambodia who had maintained stable employment in Thailand for many years and had developed long-term, trusting relationships with their employers.

The documented workers in the study had registered through the OSSC route and this usually depended on employer support. The complexity of the OSSC registration process meant that employers who wanted to be sure of their workers’ legal status needed to play an active role in guiding them through the necessary procedures.

Registration is a prerequisite for enrolment in both the MHCS and SSS, both of which entail additional costs for Thai employers acting as sponsors. Employers therefore needed to be confident that the workers concerned were reliable. Many employers covered the full cost of legal registration and MHCS enrolment for long-term employees. However, this was not the norm for newly arrived or temporary workers, for whom employers often provided only partial or no financial support:

“I need to talk to them and ask them about what they prefer to do. If the cost is too much, I need their help. I can’t subsidise all their [health] expenditure. Their preferences must be considered as well. Because they come from poor families, they want money sent back home.”

(E8)

Employers generally preferred the MHCS to the SSS. Enrolment in the MHCS is comparatively straightforward, provides one year of coverage, and requires only a single payment, which workers may help to cover. By contrast, the SSS requires ongoing employer payroll contributions of 1.5 per cent of wages and additional paperwork to terminate enrolment. It places responsibility for reimbursement claims on workers—an administrative burden that few migrants can manage independently. Moreover, most migrant workers do not benefit from the SSS pension component, as they are unlikely to remain in Thailand long enough to claim it. For these reasons, employers generally steered workers towards the MHCS:

“If you are already registered for a work permit and have the MHCS, you are eligible for the same medical benefit as a Thai. But if you haven’t got a card, you have to pay for everything at expensive prices and also could be arrested by the police. I think the card is worth the money for dental treatment. (…) How much does a normal dental treatment cost? The cost is 500–600 baht at a private clinic. The dental treatment cost in a public hospital is 280 baht [without the MHCS]. They should go there to get dental treatment with the MHCS [for 30 baht].”

(E1)

Only one worker discussed in the employer interviews was enrolled in the SSS.

The nature of the employer–worker relationship strongly influenced where and when migrant workers could seek healthcare. Employers who adopted a transactional approach, particularly those with high worker turnover, were generally less willing to invest time or money in arranging insurance coverage and more reluctant to allow time off for medical appointments. Conversely, workers in stable, long-term employment typically received greater financial support and more flexibility when seeking treatment:

“I can show them my generosity. Whenever they get sick, I allow them to go to a private clinic, not a hospital. If they go to a hospital, it means that they go on their own. I don’t go with them. (…) To solve the problem, I offer to take them to a [private] clinic. (…) I usually allow my employees to go to a clinic when they get sick. I will take care of them as much as I can. I am not seriously concerned about money, just that they get well.”

(E10)

Private clinics were often preferred by both employers and workers due to convenience and shorter waiting times. Even when employers permitted workers to attend public hospitals during working hours, this often resulted in wage deductions, offsetting any financial advantage of using public services:

“Actually, when they had a toothache I told them go to the hospital, but they refused. They prefer to go to a private clinic. It is possible that the private clinic’s service is faster. They always go to the clinic in the evening because they are in charge of the business during the day. Mostly, they focus on convenience. (…) In the past, when somebody got a toothache caused by wisdom tooth impaction, I told her to go to the hospital, but she didn’t. She decided to wait to go to the clinic.”

(E11)

Employers acknowledged that migrants often encountered discriminatory attitudes and brusque treatment in public healthcare facilities, regardless of whether they paid out of pocket or used public insurance. Although MHCS holders can receive dental treatment in public hospitals for only 30 baht per visit, perceived discrimination discouraged many from doing so:

“In reality, they really don’t want to use their entitlement to health services, except for a serious illness that a private clinic can’t handle. I think that the cost of medical care in a private clinic is more expensive, so they need to leave the customer satisfied.”

(E12)

Other employers similarly noted that workers were often reluctant to enrol in the MHCS or to use public hospital services:

“They sometimes don’t seem aware of their health rights… that it is different in a public hospital compared with a private dental clinic. Most of the employees who have the MHCS prefer going to a private dental clinic.”

(E5)

“Besides, most of the workers for whom I’ve bought the MHCS prefer going to a private clinic, not a hospital. If they want to go to a hospital they need to organise the time; it could make them lose time in work. One Laotian worker went there, the process was slow. (…) Sometimes they were in a queue, but they had to wait until evening.”

(E10)

Most employers adopted a transactional orientation towards migrant workers, with the level of support provided reflecting their assessment of how long migrants were likely to remain in the job. Many appeared more concerned about the administrative complexity of the regularisation process and enrolment in insurance schemes than with equitable dental care. Several employers reported avoiding the inconvenience of the treatment pathways provided by the MHCS by directing workers to private clinics, which were perceived as a good-enough alternative. Employers generally agreed that migrant public health insurance was beneficial, yet many questioned whether migrants had sufficient incentives to enrol. However, even where employers described their willingness to pay for treatment at private clinics as an expression of generosity towards workers, this generally served their own instrumental interests by reducing the duration of workers’ absence from work.

### 3.4. Migrant Workers

Almost all focus group participants reported experiencing oral health problems, such as mouth sores, toothache, bleeding gums, dental caries, discoloured teeth, and tooth sensitivity. Despite what appeared to be poor oral health status, participants appeared largely unconcerned. For many, an acute episode—such as an accident, loss of a filling, or severe toothache—was required to prompt a decision to seek dental care.

Most participants who had received recent dental treatment reported doing so at a public hospital or private clinic in Thailand., as opposed to their country of origin. Many described a willingness to tolerate pain or discomfort before considering a dental visit:

“I didn’t see a dentist because it was not a severe pain. My gum only swelled up for just one night. I ignored my pain. I didn’t take any pain killers or tell my employer. If I went to the hospital, the dental staff would have told me that it wasn’t a big problem.”

(UB13, age 22)

“I don’t want to go even if I have the money. I am not scared. I really don’t want to go… it is not convenient. The pain will subside by itself. I can take care of myself.”

(NP31, age 18)

Most participants stated that they would not inform their employer about what they perceived as minor or trivial symptoms.

Workers confirmed that many employers contributed to treatment costs, either fully or partially. However, they also noted that loss of wages due to absence during working hours was common. This led some respondents to seek care at private clinics outside working hours, although very few used private services without employer financial support.

Two participants reported returning to their home countries for dental treatment due to lower costs:

“Once when I had toothache I went home (to Laos) to have the tooth out after taking medicine didn’t work. I paid about 200 baht (£4.70 GBP). I have never been to a hospital or a private dental clinic in Thailand.”

(UB23, age 24)

“I always see a dentist in Cambodia because the cost of treatment in Thailand is expensive… If I get the treatment in Thailand, I would pay about 800–900 baht (£18–£21 GBP), whereas the treatment cost in Cambodia is only 150 baht (£3.50 GBP).”

(S12, age 42)

This pattern of “transnational health-seeking” has been documented in other border regions [23,24], and highlights the continuing importance of cross-border social ties and care networks.

Financially constrained workers in insecure employment appeared to weigh need, cost, and perceived benefit so as to manage the occasional dental flare-up well enough to maintain employment and continue sending remittances home.

Most migrant participants had only basic knowledge of public health insurance, and many were uncertain about their entitlement to dental care. Unregistered workers tended to be less well informed than those already enrolled. Most insured participants were covered under the MHCS and several corroborated employers’ comments regarding the unpopularity of the regular payroll deductions required by the SSS. Because the MHCS required a one-off annual payment, some younger workers declined to enrol when employers did not cover this cost, believing themselves to be in good health or perceiving treatment in their home countries to be cheaper. One participant expressed indifference about enrolment in the MHCS because his employer paid for all treatment costs:

“I am indifferent as to whether or not I have the health insurance because my boss sponsors the bills and everything… He pays for everything, even if it is only 10 or 20 baht.”

(M23, age 22)

Most workers relied on employer help for completing employment registration, and in some cases seemed to think it was the employer’s responsibility to ensure that this was completed. This might take time. Some unregistered workers described being in a liminal “betwixt and between” state, delaying dental care in anticipation of more affordable access after registration:

“I would go for tooth scaling, but my friend told me: don’t rush, just wait for the registration.”

(NP14, age 19)

Despite these challenges, most participants viewed registration and access to public insurance positively, mentioning the financial security and protection from unaffordable charges that insurance provided:

“The MHCS is good. We should have it… In the past, I didn’t have the MHCS and had a motorcycle accident… The out-of-pocket cost was 17,000 baht (£395 GBP). I had to take out a loan.”

(NK22, age 26)

“If I didn’t have the MHCS, I’d be afraid to see a dentist… I guess the medical bill might be 1000 baht.”

(NK14, age 20)

“I have the MHCS so I only pay 30 baht.”

(S12, age 42)

Overall, most workers felt that the MHCS should be retained, rather than replaced by mandatory enrolment in the SSS, which was perceived as more expensive and less flexible.

Migrant workers’ accounts of barriers to accessing public healthcare echoed concerns raised by dental professionals and employers, including long queues, lack of immediate appointments, difficulty navigating hospitals due to unfamiliar language, and perceived staff rudeness. Two undocumented participants expressed reluctance to visit hospitals staffed by Thai civil servants, likely due to concerns about their immigration status being scrutinised.

When asked about problems with registration, workers expressed much the same concerns that employers had mentioned. They emphasised the need for greater consistency in immigration regulations, a less complex registration process, and a return to the original concept of a one-stop registration service. At the time of fieldwork, workers undertaking registration were required to travel from the OSSC to local hospitals for medical examination, meaning more than a single stop. Also, a recent temporary suspension of new worker registration at OSSCs was widely criticised, as was the need to travel long distances to Bangkok or Rayong for nationality verification.

A noteworthy feature of migrants’ accounts was the extent to which their responses to dental problems involved active decision-making rather than passive compliance with suggestions from employers or the public hospital treatment pathway associated with the MHCS. These decisions were often shaped through interactions with employers, but in other cases the management of dental problems appeared to be determined by individual preferences and knowledge of the perceived costs and benefits of using public hospitals, private clinics or dental services across the border. Although some accounts may have overstated the degree of agency involved, the reported use of non-standard pathways to treatment does not readily appear to be explained by social desirability bias. Rather, these accounts suggest that many migrant workers perceive themselves as having some scope to make choices about how and where to seek dental care, despite the structural constraints they face. Overall, the focus group data suggest that migrant workers engage in active coping strategies that mediate the effects of their disadvantaged position and the limitations of public dental services.

## 4. Discussion

Our findings from 2016–2017 show that each stakeholder group—policymakers, administrators, dental professionals, employers, and migrant workers—approached dental care for migrants through a distinct but ultimately convergent logic. These perspectives differ in what is regarded as important, but together they reinforced a system in which dental care remained peripheral and under-prioritised. Policymakers and administrators tended to view current arrangements as an acceptable interim solution until greater enrolment in the SSS can be achieved, while a smaller group put forward a rights-based argument for expanding entitlements but lacked the institutional leverage to bring about change. Dental professionals, for their part, framed migrants as a challenging patient group whose needs were managed through ad hoc workarounds rather than systemic reform. Employers adopted a transactional stance, weighing costs and convenience, and often shifting responsibility for insurance uptake onto workers. Migrant workers, managing precarious finances, making commonsense calculations about need, cost and benefits, sometimes negotiating the best way forward with employers, and often delaying care until symptoms become acute. We believe that these attitudes and ways of seeing are likely to remain relevant and substantially shape the present situation.

An important contribution of this study is to show how these orientations intersect to produce what we term a confluence of instrumentality. Across all stakeholder groups, decisions are shaped by calculations of cost, convenience, and perceived need. This shared instrumental reasoning reduces the impetus for reform and helps explain why dental care—despite its importance for wellbeing, productivity, and early disease detection—remains marginal within migrant health policy. Migrants’ coping strategies, themselves shaped by insecure employment and limited entitlements, may inadvertently reinforce the status quo, even though they are rational responses to the constraints they face.

The findings align with commentary on general migrant healthcare in Thailand, including recent commentary that highlights the fragmented nature of entitlements, the administrative complexity of registration pathways, and the limited uptake of voluntary insurance schemes [1,11,25]. However, our study extends this literature by showing how stakeholders’ reasoning actively reproduces these structural problems. For example, many policymakers justified the low-priority given to migrant health, by arguing that workers would eventually transition to the SSS. Yet this assumption overlooks the reality that large numbers of migrants remain unconvinced of the benefits of insurance and unwilling to enrol due to anticipated wage deductions, probable future mobility, and possible employer non-compliance.

Dental professionals similarly adopted an instrumental stance, often describing migrants as a patient group associated with low reimbursement rates, limited coverage, and reluctance to seek care. Several dentists attributed low utilisation to migrants’ supposed unwillingness to pay or their lack of health knowledge, despite evidence that financial constraints, employer restrictions, and administrative barriers have greater impact. These perceptions echo findings from other research documenting negative stereotypes of migrants among staff in the wider healthcare system [26].

Employers’ reasoning was shaped by labour market dynamics. High turnover, uncertainty about workers’ long-term intentions and the administrative burden of registration led many employers to limit their support for insurance enrolment. This finding is consistent with broader analyses of Southeast Asian labour regimes, which emphasise the instrumental treatment of migrant workers as a flexible, expendable workforce [8]. Employers’ preference for the MHCS over the SSS, due to lower costs, simpler administration, and the absence of long-term obligations, further entrenched a system in which migrants received only basic dental coverage.

Migrant workers themselves also expressed instrumental reasoning, shaped by low wages, limited entitlements, and the need to maximise remittances. A central theme was the economic calculus that shaped decisions about when and where to seek care. Many normalised dental pain, sought care only when symptoms became severe, or delayed treatment while awaiting registration. Such behaviour is consistent with other recent research showing that migrants often prioritise immediate income over preventive health care [5].

Knowledge of public health insurance schemes was limited and uneven, particularly among unregistered workers. Although the MHCS was generally viewed positively, misconceptions about entitlements and cost-sharing were widespread. Most were uncertain about the quality of care and the staff attitudes they would encounter in public hospitals. Younger workers, in particular, expressed reluctance to pay the annual premium when employers did not subsidise it, believing themselves to be healthy or preferring to seek care in their home countries. These findings echo recent studies which show that migrants’ engagement with insurance schemes is shaped not only by affordability but also by perceived value and trust in the system [27,28], the clarity of information provided by employers or government authorities [29,30,31], and the likelihood of discriminatory behaviour by healthcare personnel [32]. These studies of the attitudes and practices of service providers and migrants in the domain of general healthcare suggest that little change has occurred in the last ten years; the same is likely to be true for dental services which are often provided in the same public general hospitals.

The concept of a confluence of instrumentality adds a new dimension to this literature by foregrounding the instrumental orientations of multiple stakeholders—policymakers, employers, health professionals, and migrants themselves. Existing studies tend to focus on structural barriers—documentation, cost, administrative hurdles, and service availability. While these are important, they do not fully explain the persistent neglect of dental care, a domain often deprioritised even within migrant-inclusive health programmes. This study highlights how migrants, too, may adopt instrumental reasoning—prioritising immediate income, avoiding wage deductions, or minimising interactions with state authorities—thereby unintentionally reinforcing existing service deficits. It captures how diverse actors, each operating under different constraints, converge on a shared outcome: the systematic de-prioritisation of dental treatments for migrant workers.

One point that deserves emphasis is that agency within constraints should not be equated with individual responsibility for social disadvantage. In this respect, our position differs from Oscar Lewis’s controversial “culture of poverty” thesis [33], which has been criticised both for neglecting the structural determinants of poverty [34] and for a cultural determinism that underplays human agency [35]. Some workers may well have developed a present-oriented approach to decision-making, prioritising immediate concerns such as maintaining paid employment, managing living costs and sending remittances over longer-term health maintenance through dental care. However, such a pattern should not be generalised or interpreted as a cultural disposition. Rather, our analysis emphasises the active coping strategies of workers who have taken the radical step of moving to another country in an attempt to improve their circumstances. Migrant workers may display considerable ingenuity in navigating the structural, legal and economic constraints they encounter and in making pragmatic choices among competing demands. Although these strategies have not yet translated into sustained pressure for improved dental services, they can be understood as rational responses to precarious employment, limited entitlements and the immediate demands of everyday survival.

Several limitations of the research should be noted. The substantial gap between the date of fieldwork and publication of the findings is clearly unfortunate. Nevertheless, we argue that the findings retain relevance because the institutional and regulatory framework has remained substantially unchanged, and studies of migrant access to general healthcare cited earlier suggest that behaviour and attitudes have also remained much the same.

The fact that the study examines policymakers and administrators, professionals, employers and migrant workers is both a strength and a limitation. The research provides a synoptic picture of the wider landscape of health services for migrant workers, but investigation of each stakeholder group rests on a modest data set. With the limited resources available, 61 in-depth interviews and 11 focus groups was as much as was feasible in a study that was not supported by external funding. Establishing data saturation is difficult when 11 focus groups, each comprising only three or four participants, are distributed across a wide geographical area. In an exploratory doctoral study of this kind, however, the scope for increasing confidence in the adequacy and diversity of the data through additional focus groups is inevitably constrained by the resources available to the project. Recruitment of both employers and migrant workers relied on convenience sampling and participants’ willingness to take part, which is likely to have resulted in the under-representation of employers with potentially sensitive issues to conceal and workers in the most precarious positions. The recruitment of migrant workers to focus groups may also have been influenced by employer gatekeeper bias, while social desirability and disclosure biases may have shaped participants’ accounts. Translation may not always have fully captured the nuances of participants’ original accounts, particularly in the two focus groups conducted predominantly in Khmer, which also involved a second translation into English with possible additional complications. Because our findings come from an exploratory case study, they should be regarded as tentative and as requiring confirmation through further research. None of the migrant workers in the present study had entered Thailand through the MOU route, which could be usefully examined in future studies.

A final limitation is that the findings cannot be confidently generalised beyond Thailand’s northeastern region. The case study describes an important route of labour migration from Laos and Cambodia, but other migration corridors have different characteristics. For example, many migrants move directly into Thailand’s central region, while those from Myanmar often work in the fishing industry or large manufacturing plants on the eastern seaboard.

## 5. Conclusions

Overall, the study confirms that migrant workers’ access to dental care is constrained by many of the structural barriers identified in studies of the wider healthcare system. Structural constraints matter, but our research shows that their effects are mediated by migrants’ perceptions and practical decisions. We argue that policy interventions will be more effective if closer attention is paid to how migrants themselves interpret and respond to these constraints, manage health problems, and navigate pathways to treatment. The decisions of all key stakeholder groups, including migrant workers, are shaped by pragmatic assessments of perceived costs and benefits. A more precise understanding of these decision-making processes may therefore identify opportunities for policy adjustment that are more feasible than comprehensive system reform. In particular, greater attention to migrants’ own accounts of why they do not follow official regularisation pathways or enrol in public insurance schemes could reveal key barriers needing early attention. Understanding the specific difficulties migrants encounter during registration, and what aspects of the MHCS or SSS make enrolment unattractive, may point to relatively small adjustments with potentially significant effects on access to care. The increase in the annual SSS dental treatment cost ceiling in May 2026 is an example of a limited policy change that may help to encourage enrolment. However, this adjustment appears to be a response to concerns raised by dentists about inadequate reimbursement rather than to migrant workers’ concerns about issues such as delay before coverage begins and the absence of effective mechanisms to address employer reluctance to pay SSS contributions. Recognising that the effects of structural constraints are mediated by migrants’ pragmatic decision-making also points to a role for measures that might strengthen their capacity to make informed choices, for example by improving migrant health literacy and developing the cultural competence of healthcare providers.

## Figures and Tables

**Table 1:** Recent incremental changes in policy framework.

Period	Policy Change	Effect on Migrants
**2019**	Minor changes in OSSC foreign-worker health examination	Examination still does not include dental health status
**2020–2022**	COVID-related extensions to regularisation	Prevented mass loss of legal status and health protection
**2022–2023**	Expansion in scale of registration/regularisation capacity	Expanded pathways for regularisation
**2024**	Additional improvements in registration of irregular workers from Laos, Cambodia, Myanmar and Vietnam	Further expanded regularisation
**2025**	Extensions of time-limited work permits Updated MOU with Cambodia	Reduced risk of workers falling out of the system Simplified MOU recruitment and introduced greater flexibility for seasonal/short-term work
**2026**	Improved SSS dental coverage so that treatments are based on need rather than an annual 900 baht cost ceiling.	Relaxation of cost threshold benefits both eligible Thais and migrant workers

**Table 2:** Study participants.

Group		Participants	
		Role	N
Policymakers and administrators	Senior officials in the Ministry of Public Health (Bangkok)	Policy development regarding healthcare for migrants workers	2
	Senior officers at NHSO (Headquarters, Bangkok)	Officials responsible for administering the UHC scheme for Thai citizens	2
	Senior officials in the NHSO regional offices in NE Thailand	Officials responsible for monitoring performance and allocating funding to contracted units for primary care (CUPs) at local level	2
	Senior officials in Provincial Health Offices (PHOs)	Officials responsible for implementing dental policy, managing human resources in hospitals, and overseeing the referral networks.	5
Dental professionals	State dental professionals and dental nurses	Dentists and dental nurses providing public sector dental services.	23
	Private dentists	Dentists providing dental services in private clinics.	6
Employers	Thai employers	Employers owning businesses employing migrant workers	21
Migrant workers	Migrant workers	Registered and undocumented migrant workers from Lao PDR and Cambodia employed in 5 provinces	11 focus groups 37 Participants

**Table 3:** Employers Interviewed.

Province	ID Code	Gender	Age	Type of Business/Shop	Number of Employees			Years in Present Position
					Thai	Cambodian	Laotian	
Nong Khai	E1	Female	42	Restaurant	3	-	3	15
	E2	Female	41	Shoe retailer	1	-	1	10
	E3	Female	40	Cosmetics and miscellaneous supplies retailer	1	-	1	18
	E4	Male	32	Vietnam coffee shop	4	-	1	2
Nakhon Phanom	E5	Male	63	Construction materials business	20	-	5	30
	E6	Male	58	Drug store	5	-	1	33
	E7	Male	57	Religious supplies retailer	1	-	2	20
	E8	Male	51	Clothes retailer	-	-	3	14
	E9	Male	48	Agricultural supplies business	-	-	3	30
Mukdahan	E10	Male	48	Electrical equipment retailer	-	-	3	10
	E11	Female	47	Toy shop	-	-	1	20
	E12	Male	31	Clothes and miscellaneous items	2	-	1	5
	E13	Female	26	Restaurant	10	-	19	40
Ubon Ratchathani, (Chong Ann Mah)	E14	Female	57	Takeaway food	-	1	-	3
	E15	Female	38	Transport company maintenance dept.	8	-	3	20
(Chong Mek)	E16	Female	47	Takeaway food	-	-	3	10
	E17	Female	44	Thai snacks	1	-	1	19
Surin, (Chong Chom)	E18	Female	38	Traditional Thai-Isaan food restaurant	2	2	-	8
	E19	Male	38	Agricultural supplies wholesaler	-	18	-	2
	E20	Female	38	Wooden furniture retailer	2	2	-	8
	E21	Female	35	Cooked-to-order food	-	5	-	6

**Table 4:** Migrant Worker Participants in 11 Focus Groups.

Province/FGD	Code	Gender	Age	Nationality	Job Position	Experience	Registration	Insurance	Dependents	Duration
Nong Khai (FGD1)	NK11	M	23	Laos	Waiter (ENI)	7	Yes	MHCS	-	21:00
Nong Khai (FGD1)	NK12	M	24	Laos	Waiter (ENI)	7	Yes	MHCS	-	
Nong Khai (FGD1)	NK13	M	22	Laos	Waiter (ENI)	2	Yes	MHCS	-	
Nong Khai (FGD1)	NK14	F	20	Laos	Waiter (ENI)	2	Yes	MHCS	-	
Nong Khai (FGD2)	NK21	M	22	Laos	Salesperson, toy shop (ENI)	2	Yes	MHCS	-	13:17
Nong Khai (FGD2)	NK22	F	26	Laos	Salesperson, toy shop (ENI)	5	Yes	MHCS	-	
Nong Khai (FGD2)	NK23	F	24	Laos	Salesperson, toy shop (ENI)	4	Yes	MHCS	-	
Nakhon Phanom (FGD1)	NP11	M	18	Laos	Petrol station attendant (ENI)	6 months	No	No	-	13:29
Nakhon Phanom (FGD1)	NP12	F	18	Laos	Petrol station attendant (ENI)	1	No	No	-	
Nakhon Phanom (FGD1)	NP13	F	20	Laos	Petrol station attendant (ENI)	6 months	No	No	-	
Nakhon Phanom (FGD1)	NP14	M	19	Laos	Petrol station attendant (ENI)	1	No	No	-	
Nakhon Phanom (FGD2)	NP21	M	44	Laos	Driver at construction materials company (E5)	20	Yes	MHCS	1	24:10
Nakhon Phanom (FGD2)	NP22	M	34	Laos	Mechanic at construction materials company (E5)	6	Yes	MHCS	-	
Nakhon Phanom (FGD2)	NP23	M	30	Laos	Salesperson at construction materials company (E5)	6	Yes	MHCS	-	
Nakhon Phanom (FGD3)	NP31	M	18	Laos	Salesperson at agricultural supplies business (E9)	3	No	MHCS	-	9:51
Nakhon Phanom (FGD3)	NP32	M	19	Laos	Salesperson at agricultural supplies business (E9)	6 months	No	No	-	
Nakhon Phanom (FGD3)	NP33	M	18	Laos	Salesperson at agricultural supplies business (E9)	1	No	No	-	
Mukdahan (FGD1)	M11	F	20	Laos	Waiter (E13)	3 months	No	No	-	31:00
Mukdahan (FGD1)	M12	F	19	Laos	Waiter (E13)	3 months	No	No	-	
Mukdahan (FGD1)	M13	F	20	Laos	Waiter (E13)	8 months	No	No	-	
Mukdahan (FGD1)	M14	F	22	Laos	Waiter (E13)	1 yr 3 months	No	No	-	
Mukdahan (FGD2)	M21	F	20	Laos	Salesperson at electrical equipment retailer (E10)	1	Yes	MHCS	-	18:49
Mukdahan (FGD2)	M22	F	18	Laos	Salesperson at electrical equipment retailer (E10)	1	Yes	MHCS	-	
Mukdahan (FGD2)	M23	F	22	Laos	Salesperson at electrical equipment retailer (E10)	2	Yes	MHCS	-	
Ubon Ratchathani (Chong Ann Mah)	UB11	M	22	Laos	Transport company maintenance dept. (E15)	4	Yes	MHCS	-	19:28
Ubon Ratchathani (Chong Ann Mah)	UB12	M	23	Laos	Transport company maintenance dept. (E15)	4	Yes	MHCS	-	
Ubon Ratchathani (Chong Ann Mah)	UB13	M	22	Laos	Transport company maintenance dept. (E15)	4	Yes	MHCS	-	
Ubon Ratchathani (Chong Mek)	UB21	F	19	Laos	Cook, take-away food (E16)	1	No	No	-	14:01
Ubon Ratchathani (Chong Mek)	UB22	F	22	Laos	Cook, take-away food (E16)	3	No	No	-	
Ubon Ratchathani (Chong Mek)	UB23	F	24	Laos	Cook, take-away food (E16)	3	No	MHCS	-	
Surin (FGD1)	S11	F	32	Cambodian	Salesperson at fruit wholesaler (ENI)	4	Yes	MHCS	-	19:17
Surin (FGD1)	S12	F	42	Cambodian	Salesperson at fruit wholesaler (ENI)	3	Yes	MHCS	-	
Surin (FGD1)	S13	F	38	Cambodian	Salesperson at fruit wholesaler (ENI)	1	Yes	MHCS	1	
Surin (FGD1)	S14	F	22	Cambodian	Salesperson at fruit wholesaler (ENI)	2	Yes	No	-	
Surin (FGD2)	S21	M	47	Cambodian	Ironing staff at second-hand clothes retailer (ENI)	4	Yes	No	1	17:19
Surin (FGD2)	S22	F	21	Cambodian	Salesperson at wooden furniture retailer (E20)	3	Yes	No	-	
Surin (FGD2)	S23	F	23	Cambodian	Salesperson at wooden furniture retailer (E20)	3	Yes	No	1	

No = 37. ENI = Employer not interviewed; otherwise the employer ID is provided. “Chong” (in Thai) means border crossing area.

## Data Availability

The data presented in this study are available on request from the corresponding author, subject to limitations required by the research ethics committee to preserve participant confidentiality. In accordance with the conditions of REC approval, audio recordings have been destroyed, and the authors are unable to supply full interview or focus group transcripts.
